# Stratification substantially reduces behavioral variability in the hypoxic–ischemic stroke model

**DOI:** 10.1002/brb3.77

**Published:** 2012-07-16

**Authors:** Julia Pollak, Kristian P Doyle, Lauren Mamer, Mehrdad Shamloo, Marion S Buckwalter

**Affiliations:** 1 Department of Neurology and Neurological Sciences, Stanford University School of MedicineStanford, California; 2 Department of Neurosurgery, Stanford University School of MedicineStanford, California; 3 Stanford Behavioral and Functional Neuroscience Laboratory, Stanford Institute for Neuro-Innovation and Translational Neurosciences, Stanford University School of MedicineStanford, California

**Keywords:** Behavior, hypoxic–ischemic stroke, motor recovery, mouse model

## Abstract

Stroke is the most common cause of long-term disability, and there are no known drug therapies to improve recovery after stroke. To understand how successful recovery occurs, dissect candidate molecular pathways, and test new therapies, there is a need for multiple distinct mouse stroke models, in which the parameters of recovery after stroke are well defined. Hypoxic–ischemic stroke is a well-established stroke model, but behavioral recovery in this model is not well described. We therefore examined a panel of behavioral tests to see whether they could be used to quantify functional recovery after hypoxic–ischemic stroke. We found that in C57BL/6J mice this stroke model produces high mortality (approximately one-third) and variable stroke sizes, but is fast and easy to perform on a large number of mice. Horizontal ladder test performance on day 1 after stroke was highly and reproducibly correlated with stroke size (*P* < 0.0001, *R*^*2*^ = 0.7652), and allowed for functional stratification of mice into a group with >18% foot faults and 2.1-fold larger strokes. This group exhibited significant functional deficits for as long as 3 weeks on the horizontal ladder test and through the last day of testing on automated gait analysis (33 days), rotarod (30 days), and elevated body swing test (EBST) (36 days). No deficits were observed in an automated activity chamber. We conclude that stratification by horizontal ladder test performance on day 1 identifies a subset of mice in which functional recovery from hypoxic–ischemic stroke can be studied.

## Introduction

There are seven million adults in the United States, or 3% of the population, who have suffered a stroke (Roger et al. 2012). Approximately one-third of these people are physically disabled by their stroke. This results in stroke being a leading cause of serious, long-term disability in the United States. There are currently no Food and Drug Administration (FDA) approved treatments to improve functional recovery after stroke. Although molecular and cellular processes such as dendritic and axonal sprouting, neurogenesis, and inflammation are widely thought to affect recovery from stroke, our understanding of how significantly each contributes to recovery remains limited. A major barrier to expanding our understanding of stroke recovery is the dearth of standardized animal models of recovery – such models allow for confirmation of results across labs and comparison of effect sizes between treatment groups. Methods to follow poststroke recovery in mice are particularly useful since sophisticated genetic manipulations can be performed in mouse models. We, therefore, set out to develop a standardized mouse model to study recovery from stroke.

Hypoxic–ischemic stroke is a well-described model first reported in 1960 in adult rats by Levine (1960), and has been used extensively in adult rats and mice as well as in neonatal rodents. Hypoxia causes thrombosis on the side of unilateral common carotid occlusion, resulting in injury to the ipsilateral cortex, hippocampus, and striatum, but sparing the contralateral hemisphere, which exhibits normal perfusion during hypoxia (Adhami et al. 2006). We chose hypoxic–ischemic stroke because it is a high throughput model that allows behavioral testing to be performed on large groups of mice in parallel, minimizing the effects of day-to-day variability. Importantly, it results in a significant neurological injury similar to that seen in disabling human stroke. Functional deficits have been reported in this model (Olson et al. 2004; Olson and McKeon 2004; Guzman et al. 2008; Andres et al. 2011) but so has stroke size variability (Kuan et al. 2003; Olson et al. 2004; Adhami et al. 2006).

We, therefore, chose to develop a model for studying functional recovery after hypoxic–ischemic stroke in C57BL/6J mice, and investigated how stroke size variability affected a panel of functional tests in the weeks after hypoxic–ischemic stroke. C57BL/6J is the most commonly used mouse strain for both stroke and genetic models. We found that the hypoxic–ischemic stroke procedure was consistent between surgeons. The model did cause variable stroke size in our hands, ranging from negligible to large enough to cause fatality. We found that functional testing on day 1 after stroke with a simple and inexpensive horizontal ladder test (Metz and Whishaw 2002) can be used to define a set of mice with large, relatively homogeneous strokes that are suitable for long-term studies of functional recovery. In this group of mice, we detected deficits in horizontal ladder, automated gait analysis/Catwalk, rotarod, and elevated body swing test (EBST) that lasted for weeks. The ladder and Catwalk tests could both be used to follow recovery for 3–5 weeks after stroke, and the rotarod and EBST tests demonstrated a fixed deficit that did not improve over the 5 weeks of testing after stroke. Activity chamber testing did not record deficits after hypoxic–ischemic stroke.

## Materials and Methods

### Animals

All animal procedures were reviewed and approved by the Stanford University Institutional Animal Care and Use Committee. C57BL/6J male mice (Jackson Laboratories, Bar Harbor, ME) were 5 months old at the time of surgery and were used for all studies except 2,3,5-triphenyltetrazolium chloride (TTC) staining, which was performed in 2-month-old female albino C57BL/6J mice.

### Hypoxia–ischemia model

Mice were anesthetized by 2% isoflurane in 100% oxygen. An incision was made in the neck to expose the common carotid arteries and the right common carotid artery was permanently ligated. After a recovery period of approximately 2 h, mice were placed in a plastic chamber containing 8% oxygen and 92% nitrogen for 25 min. The chamber was a standard mouse cage without bedding (26 × 18 × 2.5 cm), and was partially submerged in a 37°C water bath to maintain normothermia during hypoxia. Fresh gas flowed continuously into the chamber throughout hypoxia through vents in the chamber lid. Following hypoxia mice were kept in a warm cage to maintain normothermia and had free access to soft food and water. Sham animals underwent surgical dissection of the right carotid artery, without carotid ligation or hypoxia.

### Stroke size

To assess stroke size and location in our hands, we first performed TTC staining 24 h after hypoxic–ischemic stroke. To assess stroke size in surviving mice and determine whether stroke variability was due to differences in surgical technique, two surgeons performed hypoxic–ischemic strokes in a larger group of 42 mice.

### Experimental design

**Figure 1:**
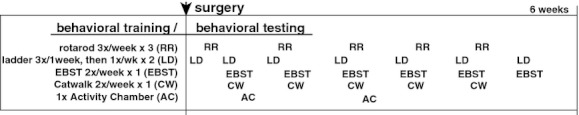
Experimental design. Mice were trained and tested as shown, and sacrificed for stroke volumes 6 weeks after stroke.

Mice were shipped from The Jackson Laboratories and acclimated to our mouse room for at least 1 week. They were then handled multiple times and most often sat calmly on the experimenter's hand when removed from their cage. Mice were trained on all behavior tests prior to surgery, then tested at the intervals shown (Fig. 1). All behavior tests were performed in the light cycle and at the same time of day on each day of testing. All apparatuses were disinfected (with Simple Green (Huntington Beach, California) unless otherwise noted) between trials, and all tests and analyses performed by an evaluator blinded to experimental groupings.

### Rotarod

Motor coordination was tested using the rotarod apparatus on days 2, 9, 16, 23, 30, and 37 post surgery. Animals were placed on a ROTOR-ROD™ (San Diego Instrument, San Diego, CA; lane dimensions 11.4 cm wide per animal; rod diameter 3.2 cm) that accelerated from 5 to 10 rpm over 300 sec, and the latency to fall was automatically recorded. Trials were ended and recorded if mice clung to the rod without walking for two revolutions. Three trials were performed on each testing day, and animals received at minimum 30 min of rest between trials. Presurgery training consisted of 12 days of testing, and the average of the last three training days before surgery was considered baseline average. Animals that did not reach 250 sec by the end of presurgery training were excluded from the study.

### Ladder test

The ladder test was adapted from the ladder rung walking task (Metz and Whishaw 2002; Farr et al. 2006). The apparatus consisted of two plexiglass walls (76.2 × 0.635 × 15.24 cm) spaced 3.175 cm apart, just wide enough for the animal to pass. Plexiglass rungs (10.16 cm long with a 0.3175 cm diameter) were inserted across the length of the walls and spaced at a constant distance of 0.635 cm apart. Both ends of the apparatus were placed atop standard mouse housing cages so that the rungs were 38 cm from the tabletop. A desk lamp illuminating the start zone incentivized the animals to traverse the ladder, and a small igloo hide leading to the home cage was placed at the end zone.

The test was performed on days 1, 4, 7, 14, 21, 28, and 35 post surgery. Presurgery training was necessary to train the animals to spontaneously traverse the apparatus. Seven presurgery trials were required for animals to make an acceptable minimum number of limb placing errors (<12% error). Traversing animals were recorded from below with a handheld camcorder. Each animal was tested once for a given day and each trial was analyzed frame-by-frame using standard film editing software (iMovie for Mac OSX 10.4). Tallying began once the first visible full gait cycle had been completed, that is, all four limbs had been placed. If a limb was placed on a rung and was not subsequently removed, a correct step was recorded. If a limb was placed between rungs, a missed step was recorded. If a limb was placed and subsequently replaced on the same rung, a correct step and an additional correction step were recorded. If a limb was placed and subsequently slipped off the rung, a correct step and an additional missed step were recorded. Tallying was complete after the animal's final full gait cycle. Percent error was calculated as: 100 × (missed step/[missed + correct + correction step]).

### Elevated body swing test

Animals were held 1 cm from the base of the tail and suspended 1–5 cm above a flat surface as described (Borlongan and Sanberg 1995; Shyu et al. 2004). One swing was recorded for each suspension. A swing was considered a 10% or greater deflection from body midline or rotation about the vertical axis. Animals were placed onto the surface between suspensions and allowed to reposition. Animals were resuspended once they were visibly balanced and not displaying a preference for one side. The evaluator varied the hand used to pick up the animal and the position they were standing to avoid biasing the direction of swings, and there were no objects immediately surrounding the testing arena for this reason. Twenty swings were recorded per trial, and side preference was calculated as swings to one side/total swings.

### Catwalk

Animals underwent two training days where they learned to cross the raised glass platform on the Noldus Catwalk (Noldus Information Technology, Wageningen, The Netherlands) apparatus. A third training day was recorded to obtain baseline. Mice were then tested weekly for 5 weeks following stroke on days 5, 12, 19, 26, and 33. Testing was performed in a darkened room and the animal's home cage was placed at the end zone.

### Activity chamber

Animals were placed into the activity chamber (Med Associates, St Albans, Vermont) and allowed to explore freely for 15 min. The 43.2 × 43.2 cm arena was placed within a sound-attenuated chamber, 66 × 55.9 × 55.9 cm. Movement parameters were calculated using software based on infrared beam breaks.

### TTC staining

TTC vital dye was utilized to assess stroke size at 24 h. Brains were sectioned into 1-mm-thick slices and each slice was immersed in 1.5% TTC in phosphate buffered saline (PBS) at 37°C for 15 min and then fixed in 10% formalin.

### Histology

Mice were terminally anesthetized with chloral hydrate and perfused with heparinized saline. Brains were fixed 24 h in 4% paraformaldehyde and then sunk through 30% sucrose in phosphate buffered saline. Forty micrometer coronal sections were cut sequentially into 24 tubes using a freezing sliding microtome (Leica Microsystems, Wetzlar, Germany) such that each tube represented an equally spaced sample of sections 960 *μ*m apart. One tube was stained using cresyl violet and the remaining tissue in the hemispheres ipsilateral and contralateral to stroke were traced.

### Statistics

Repeated measures analysis of variance (ANOVA), Mann–Whitney and *t*-tests (Prism 5.0 statistical software for Mac OS X) were used to analyze behavioral test results as indicated in the figure legends. Mice that were not able to complete the ladder test on day 1 (*n* = 1), or could not swing in the EBST on day 4 (*n* = 2) were given the maximum score that any mouse was given on that day.

## Results

### Hypoxic–ischemic stroke in adult C57BL/6J mice results in a variable stroke size

**Figure 2:**
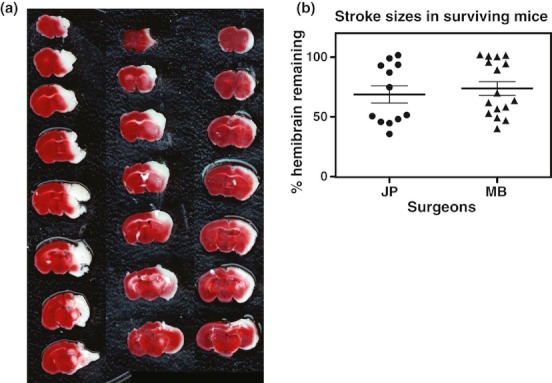
Hypoxic–ischemic stroke results in variable stroke size. (a) Typical TTC stains from 24 h after hypoxic–ischemic stroke. Left, large and likely fatal stroke; middle, survivable large stroke; right, small stroke. (b) Stroke size in surviving mice does not depend on the surgeon and most surviving mice have either very large or very small strokes. Bars, mean ± SEM.

We observed a wide variety of stroke sizes at 24 h, with three typical types of stroke. Some mice exhibited ischemia in the vast majority of the hemisphere (Fig. 2a, left), some had an intermediate-sized stroke with dense ischemia in the cortex and hippocampus and more diffuse injury in the striatum (Fig. 2a, middle), and others had either no visible ischemia or a small cortical stroke (Fig. 2a, right). Of 13 mice that underwent hypoxic–ischemic stroke, the five with the smallest strokes averaged a stroke size of 11.4 ± 2.4% of the contralateral hemisphere. Five mice had large strokes that measured 38.2 ± 2.7% of the hemisphere, and the remaining three mice had very large strokes measuring 56.4 ± 4.4% of the hemisphere. These three categories were all statistically different in size (small vs. large, *P* < 0.0001; large vs. very large, *P* < 0.001; ANOVA with Tukey's post hoc.)

In a larger cohort that we followed for 6 weeks, all-cause mortality was approximately one-third, and the majority of this was during hypoxia or during the first 3 days after stroke. In the surviving 28 mice, there was no difference in the mean or distribution of stroke sizes between surgeons (Fig. 2b). A dichotomization in stroke size was evident in the surviving mice, with one group of mice exhibiting loss of about 50% of the hemisphere and the other exhibiting much smaller stroke sizes. Although we did not autopsy mouse brains, the largest stroke size that we observed at 24 h in the smaller cohort (Fig. 2a, left) did not appear in the surviving mice and so was likely fatal.

### Horizontal ladder test performance on day 1 predicts stroke size at 6 weeks

**Figure 3:**
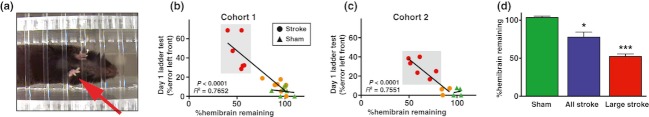
Mouse performance on the horizontal ladder test 1 day after stroke correlates with stroke size at 6 weeks after stroke. (a) Single frame shot from a video of a mouse traversing the horizontal ladder. The arrow identifies a left front paw error. (b and c) Percent error with the left front paw as measured on the ladder test correlates significantly with the percent right hemisphere remaining at 6 weeks after stroke in two separate mouse cohorts. *P* and *R* ^2^ values are from linear regression with the Stroke group mice; lines on graphs correspond to Stroke (circles) and Sham (triangles) groups. Large Stroke, red; All Stroke, red + orange. (d) Percent right hemibrain remaining in each group after stratification. Student's *t*-test ***P < 0.0001; *P < 0.05. Bars, SEM.

Given the dichotomization of stroke sizes in this model, we hypothesized that the smaller stroke sizes would result in either a quickly recovering deficit or no deficit and would introduce increased behavioral variability. In order to be able to study long-term functional recovery, our goal was to identify the subset of mice with survivable larger strokes during the first week after stroke. We examined the linear correlation between stroke size and performance in the Stroke group on the horizontal ladder test (Fig. 3a) on day 1 after stroke, rotarod on day 2, EBST on day 4, and from automated gait analysis, stride length and swing speed on day 5. Of these measures, only EBST and ladder correlated significantly among the stroked mice. Horizontal ladder performance on day 1 correlated highly with stroke size (*P* < 0.0001, *R*^*2*^ = 0.7652; Fig. 3b). This was reproducible in a second cohort of mice (*P* < 0.0001, *R*^*2*^ = 0.7551; Fig. 3c).

EBST on day 4 also correlated with stroke size, but not as tightly as horizontal ladder testing (*P* = 0.0061, *R*^*2*^ = 0.4785). Rotarod on day 2 correlated significantly only when the sham mice were added to the correlation (*P* = 0.0352, *R*^*2*^ = 0.2237).

We also examined interrater reliability on horizontal ladder test scoring. Two blinded raters (KD and LM) examined videos from 32 mice that were tested on day 1 after hypoxic–ischemic stroke. Interrater reliability was excellent, with Spearman's coefficient 0.873 (*P* = 7.5 × 10^−11^).

Based on the linear correlation between stroke size and day 1 horizontal ladder performance, we chose a cutoff of >18% error with the left front foot to assign mice to a “Large Stroke” group (Fig. 3b and c, gray box). In comparison to all stroked mice (“All Stroke”), this resulted in groupings of mice where the remaining right hemisphere volume, expressed as a percent of left hemisphere volume, was 52.3 ± 3.3%, *n* = 6 in “Large Stroke”; 77.6 ± 6.6%, *n* = 14 in “All Stroke”; and 103.6 ± 1.8%, *n* = 6 in “Sham” (Fig. 3d). The “Large Stroke” group had less variability and also was more significantly different from sham mice than the “All Stroke” group. Left hemisphere size was not different in stroked mice than in sham mice (data not shown), supporting others' data that the hypoxic–ischemic stroke model does not cause significant ischemic damage to the contralateral hemisphere in C57BL/6J mice (Kuan et al. 2003; Olson et al. 2004; Adhami et al. 2006).

### Stroke-induced spontaneous gait and gait accuracy deficits demonstrated recovery over the course of the study

We evaluated two measures of spontaneous gait after hypoxic–ischemic stroke. The first was horizontal ladder test performance, which measures limb placement errors on a ladder and which we used for identification of the “Large Stroke” group (Fig. 3). The second measure was automated gait analysis using a Catwalk (Noldus) apparatus. In both cases the mouse is walking toward its home cage at a normal speed.

**Figure 4:**
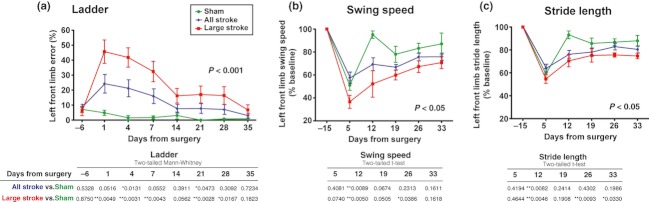
Gait measures demonstrate stroke-induced deficits that recover during the weeks after hypoxic–ischemic stroke. (a) Horizontal ladder testing and statistics. Left front swing speed (b) and stride length (c) from automated gait analysis. Bars, SEM; *P* values on graphs are repeated measures ANOVA for Large Stroke versus Sham over the course of the study; tables below the graphs are results for each day using *t*-test for parametric data and Mann–Whitney for nonparametric, as indicated.

Horizontal ladder testing was done on days 1, 4, and 7 and then weekly until day 35 after stroke (Fig. 4a). We examined foot faults with all limbs and found that the front limb contralateral to the stroke, the left front, was the most reliable to measure. Right front foot faults and bilateral hind limb faults did not change after stroke. Ladder performance in the “All Stroke” group did worsen after stroke, but was only significant on days 4 and 21 (Fig. 4a). The “Large Stroke” group displayed significantly worse function on all days except 14 and 35.

Automated gait analysis yielded many measures, most of which demonstrated some changes after stroke. No measure was different between groups before surgery. We chose to focus on stride length and swing speed because they displayed statistically significant changes after stroke, and both measures are relevant to clinical functional deficits. Segregation of mice into “Large Stroke” versus “All Stroke” groups was not necessary to see differences on day 12 – both groups were significantly different from Sham on day 12 in both measures (Fig. 4b and c). Swing speed was also impaired on day 26 in the “Large Stroke” group, as was stride length on days 26 and 33.

### Rotarod reveals poststroke deficits that do not recover after 1 month

**Figure 5:**
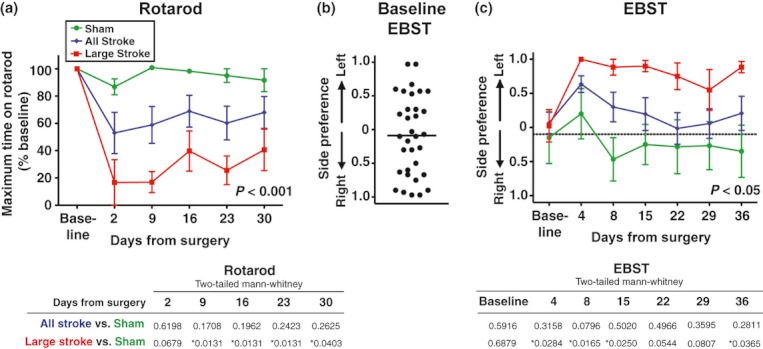
Rotarod and EBST testing deficits persist after 1 month. (a) Rotarod testing demonstrated clear deficits after stroke in the Large Stroke group, but nonsignificant deficits in the entire group. (b) Baseline EBST results reflected side preferences in mice. Each dot represents the average of three prestroke testing days. Bar, mean. (c) EBST testing demonstrates deficits in the Large Stroke group but not in the All Stroke group. Black dotted line indicates baseline average for all mice. Bars, SEM; *P* values on graphs are repeated measures ANOVA for Large Stroke versus Sham over the course of the study. Tables below the graphs are results for each day using Mann–Whitney as data were nonparametric for both tests.

We next evaluated function on the rotarod, which tests how long a mouse can remain on a rotating rod. In this study we trained mice extensively and only included mice that had learned the task before surgery (latency to fall >250 sec), so we were testing motor recovery and not motor learning. No significant differences were detectable among groups before surgery. We observed a nonstatistically significant decrease in rotarod performance in the “All Stroke” group, but segregating out the “Large Stroke” group yielded significance for all days (Fig. 5a). Mice in the “Large Stroke” group did not demonstrate significant recovery of rotarod ability over the course of the study.

### EBST testing was variable, but revealed poststroke deficits out to 5 weeks

The EBST is a measure of postural asymmetry that measures the direction animals turn toward when they are held by the tail. Interestingly, many mice exhibited a side preference on baseline testing, with the average mouse preferring to twist to the right, but all types were seen (Fig. 5b). No significant differences in side preference were detectable between surgical groups at baseline. After surgery, the “Large Stroke” group demonstrated a clear effect of stroke by preferring to swing to the contralateral side (Fig. 5c), while large variability in the “Sham” group limits the usefulness of this test. Subtracting each mouse's baseline preference did not alter the results in terms of trends or statistical significance and did decrease the variability in the shams while increasing the variability in the stroked mice (data not shown).

### There were no stroke-induced changes in spontaneous activity

**Figure 6:**
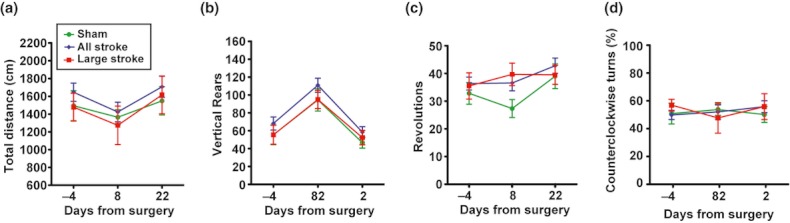
Activity chamber demonstrated no significant stroke-induced deficits. There were no differences between groups in (a) total distance traveled, (b) vertical rears, (c) total revolutions, or (d) direction of revolutions, as shown here by % counterclockwise revolutions. Bars, SEM.

To assess spontaneous activity, mice were evaluated in an activity chamber before and 8 and 22 days after stroke or sham surgery. Neither “All Stroke” nor “Large Stroke” groups exhibited differences from “Sham” mice in total distance traveled or number of vertical rears (Fig. 6a and b). The apparatus also recorded revolutions, or which way the mice turned as they explored the chamber. Despite the asymmetry observed in the Large Stroke group on EBST, there was no difference between groups in the number or direction of spontaneous revolutions (Fig. 6c and d). Finally, mice in each group spent equal proportions of their time in the periphery compared with the center of the chamber, implying that stroke did not affect anxiety levels. At baseline (day −4), the percent of time spent in the periphery of the chamber was Sham 54.9 ± 4.8% versus Large Stroke 65.4 ± 5.7%; on day 8, Sham 65.1 ± 4.6% versus Large Stroke 56.4 ± 5.8%; and on day 22, Sham 56.9 ± 6.0% versus Large Stroke 60.1 ± 5.3%.

## Discussion

To our knowledge this is the first comprehensive assessment of multiple behavioral tests, followed over time, in mice that have undergone hypoxic–ischemic stroke. Other researchers have used this model in C57BL/6J mice and reported functional deficits on rotarod out to 17 days (Guzman et al. 2008) and horizontal ladder to 4 weeks (Andres et al. 2011). Rotarod, activity chamber, and hang test deficits have also been reported at 2 days after hypoxic–ischemic stroke (Olson et al. 2004; Olson and McKeon 2004). In this study, we found that we could improve the model by using a horizontal ladder foot fault test 1 day after stroke to identify a group of mice with large strokes. Using the criteria of >18% error on the ladder test, we defined a “Large Stroke” group of mice. This group displayed significant deficits, compared with sham mice, on a panel of functional tests. The ladder test and automated gait analysis demonstrated recovery – mice improved between the first and fifth weeks after stroke, and so these tests are best used to examine rates of recovery. Rotarod and EBST demonstrated significant deficits that did not recover in the first 5 weeks after stroke, and may be the most useful tests for longer term studies. Finally, there were no deficits observed in activity chamber measures.

The hypoxic–ischemic stroke model has significant benefits as a mouse model of functional recovery. The model lends itself well to being scaled up for large groups of mice – surgical procedures are quick and require only basic surgical skills. Multiple surgeons can work in parallel because there is no difference in stroke sizes between surgeons. Additionally, for trials of prorecovery treatments mice can be sorted into equally impaired groups prior to the start of treatment, increasing the power of each test.

Another major benefit of the hypoxic–ischemic stroke model is that it is different from other commonly used models, including temporary or permanent proximal middle cerebral artery (MCA) occlusion and photothrombotic stroke. Although infarction in hypoxic–ischemic stroke is caused by MCA thrombosis (Adhami et al. 2006), this occurs in the setting of global hypoxia. Thus, there may be differences in the mechanism of cell death and/or in the neuroinflammatory response in this model compared with that seen in the more commonly used models. With the advent of new prorecovery therapies, both small molecules and stem cell treatments, there is a growing interest in ensuring that rodent studies will translate well to studies in patients. Testing new therapies in multiple, distinct, rodent models that exhibit sustained functional deficits may improve the chances of new potential therapies translating successfully to patients with stroke (STEPS Participants 2009).

However, a major disadvantage of the hypoxic–ischemic stroke model is stroke size variability. Stroke size variability results in a large proportion of mice with either fatal or negligible strokes. In the behavior study reported here, we began with 33 mice and ended with six mice in the “Sham” group and six in the “Large Stroke” group. Most of the lost mice were due to death, likely due to cerebral edema from large stroke size. We did not examine deceased mice for cerebral edema, but others have reported cerebral edema in this model (Adhami et al. 2006). Five mice were excluded prior to stroke because they failed to learn the rotarod task. The other mice “lost” out of the cohort were due to small strokes (eight of the 14 mice that survived stroke). The proportion of death and small strokes was slightly worse in this study than in later ones in our laboratory (Han et al. 2012), but we believe it is a consistent issue in this model in C57BL/6J mice.

Hypoxic–ischemic stroke size in adult C57BL/6J mice has been reported to be variable (Kuan et al. 2003; Adhami et al. 2006). Some degree of variability in the female cohort tested at 24 h (Fig. 2b) may be due to differences in estrous cycle between mice (McCullough and Hurn 2003); however, we also observed variability in the male cohort (Fig. 3b and c). Thus, the high degree of variability in stroke size may more likely be due to differences in circle of Willis anatomy between individual mice as well as other differences downstream from the point of ligation (Barone et al. 1993; Fujii et al. 1997). We have found that altering the period of hypoxia to 1 h does not alter lesion size or variability, and shorter hypoxia times result in less injury (K. P. Doyle and M. S. Buckwalter, unpubl. ms.) and so changing the length of hypoxia is not a way of refining this model.

To work around the stroke size variability in this model, we propose using the horizontal ladder test on day 1 after stroke to identify mice with larger strokes. This is inexpensive – we had our ladder constructed at a local plastics shop for under $300. The correlation between ladder results and stroke size is reproducible (Fig. 2), and test scoring exhibits excellent interrater reliability. We show here that ladder test results can be used to identify a cohort of mice with large strokes and consistent behavioral deficits. This could alternatively, and arguably more accurately, be done using MRI or CT scans. However, these imaging modalities are expensive and are not available at every center. We demonstrate here that stratification does decrease the mouse numbers needed in the beginning cohort by decreasing mouse-to-mouse variability within groups. For almost every functional outcome, we find more significant deficits in the “Large Stroke” group identified by this stratification than in the “All Stroke” group (Fig. 5.). However, even with this refinement the hypoxic–ischemic stroke model in C57BL/6J mice still requires the use of more mice than other models, such as photothrombotic motor cortex stroke, where almost all mice have identical lesions and reproducible motor deficits (Clarkson et al. 2010, 2011).

In summary, we report here a protocol for studying functional recovery after hypoxic–ischemic stroke. The surgical procedure is easy to learn and standardize, and requires no expensive equipment. Stroke size is variable, and the horizontal ladder test is a reliable indicator of stroke size 1 day after stroke. Four behavioral tests were noted to be useful in this stroke model – two that recover to baseline in young adult mice over the course of 4–5 weeks (ladder and automated gait analysis) and two that do not recover appreciably in this timeframe (rotarod and EBST).

## References

[b1] Adhami F, Liao G, Morozov YM, Schloemer A, Schmithorst VJ, Lorenz JN (2006). Cerebral ischemia-hypoxia induces intravascular coagulation and autophagy. Am. J. Pathol.

[b2] Andres RH, Choi R, Pendharkar AV, Gaeta X, Wang N, Nathan JK (2011). The CCR2/CCL2 interaction mediates the transendothelial recruitment of intravascularly delivered neural stem cells to the ischemic brain. Stroke.

[b3] Barone FC, Knudsen DJ, Nelson AH, Feuerstein GZ, Willette RN (1993). Mouse strain differences in susceptibility to cerebral ischemia are related to cerebral vascular anatomy. J. Cereb. Blood Flow Metab.

[b4] Borlongan CV, Sanberg PR (1995). Elevated body swing test: a new behavioral parameter for rats with 6-hydroxydopamine-induced hemiparkinsonism. J. Neurosci.

[b5] Clarkson AN, Huang BS, Macisaac SE, Mody I, Carmichael ST (2010). Reducing excessive GABA-mediated tonic inhibition promotes functional recovery after stroke. Nature.

[b6] Clarkson AN, Overman JJ, Zhong S, Mueller R, Lynch G, Carmichael ST (2011). AMPA receptor-induced local brain-derived neurotrophic factor signaling mediates motor recovery after stroke. J. Neurosci.

[b7] Farr TD, Liu L, Colwell KL, Whishaw IQ, Metz GA (2006). Bilateral alteration in stepping pattern after unilateral motor cortex injury: a new test strategy for analysis of skilled limb movements in neurological mouse models. J. Neurosci. Methods.

[b8] Fujii M, Hara H, Meng W, Vonsattel JP, Huang Z, Moskowitz MA (1997). Strain-related differences in susceptibility to transient forebrain ischemia in SV-129 and C57black/6 mice. Stroke.

[b9] Guzman R, De Los Angeles A, Cheshier S, Choi R, Hoang S, Liauw J (2008). Intracarotid injection of fluorescence activated cell-sorted CD49d-positive neural stem cells improves targeted cell delivery and behavior after stroke in a mouse stroke model. Stroke.

[b10] Han J, Pollak J, Yang T, Siddiqui MR, Doyle KD, Taravosh-Lahn K (2012). A small molecule TrkB ligand promotes recovery when started 3 days after stroke. Stroke.

[b11] Kuan CY, Whitmarsh AJ, Yang DD, Liao G, Schloemer AJ, Dong C (2003). A critical role of neural-specific JNK3 for ischemic apoptosis. Proc. Natl. Acad. Sci. USA.

[b12] Levine S (1960). Anoxic-ischemic encephalopathy in rats. Am. J. Pathol.

[b13] McCullough LD, Hurn PD (2003). Estrogen and ischemic neuroprotection: an integrated view. Trends Endocrinol. Metab.

[b14] Metz GA, Whishaw IQ (2002). Cortical and subcortical lesions impair skilled walking in the ladder rung walking test: a new task to evaluate fore- and hindlimb stepping, placing, and co-ordination. J. Neurosci. Methods.

[b15] Olson EE, McKeon RJ (2004). Characterization of cellular and neurological damage following unilateral hypoxia/ischemia. J. Neurol. Sci.

[b16] Olson EE, Lyuboslavsky P, Traynelis SF, McKeon RJ (2004). PAR-1 deficiency protects against neuronal damage and neurologic deficits after unilateral cerebral hypoxia/ischemia. J. Cereb. Blood Flow Metab.

[b17] Roger VL, Go AS, Lloyd-Jones DM, Benjamin EJ, Berry JD, Borden WB (2012). Heart disease and stroke statistics–2012 update: a report from the American Heart Association. Circulation.

[b18] Shyu WC, Lin SZ, Yang HI, Tzeng YS, Pang CY, Yen PS (2004). Functional recovery of stroke rats induced by granulocyte colony-stimulating factor-stimulated stem cells. Circulation.

[b19] STEPS Participants (2009). Stem Cell Therapies as an Emerging Paradigm in Stroke (STEPS): bridging basic and clinical science for cellular and neurogenic factor therapy in treating stroke. Stroke.

